# Supplementary material for: Mature MUC5AC Expression in Resected Pancreatic Ductal Adenocarcinoma Predicts Treatment Response and Outcomes
Source: Int J Mol Sci. 2024 Aug 20;25(16):9041. doi: 10.3390/ijms25169041 (PMC11354508; doi:10.3390/ijms25169041)
Supplement: Supplementary file 1 [file ijms-25-09041-s001.zip › ijms-3117495-supplementary.pdf]

Supplementary tables

Supplementary Table S1: Distribution of MUC5AC expression in the tested samples

	Mature		Immature
	Detection rate in %/H-score median (range)/H-score mean		
Population (N)	45M1	EC-45M1*	CLH2
Total (100)	96%/150(0-300)/148.5	72%	96%/150(0-300)/145.2
NAT (43)	91%/120(0-270)/129	58%	91%/100(0-270)/122
UpS (57)	100%/180 (0-300)/164	82%	100%/180(0-300)/163
NAT vs. UpS (p-value)	*0.06*	**0.007**	**0.02**

*Detection rates, NAT- neoadjuvant therapy, UpS – upfront surgery

Supplementary Table S2: Comparison of intracellular MUC5AC in extracellular-positive and -negative patients

		Extracellular		p-value
		Positive	Negative	
All patients	45M1	183	59	<0.001
	CLH2	179	59	
NAT-group	45M1	185	50	<0.001
	CLH2	174	50	
UpS-group	45M1	183	75	<0.001
	CLH2	181	77	

NAT- neoadjuvant therapy, UpS – upfront surgery.

Supplementary Table S3: Univariate logistic regression model for MUC5AC expression in the neoadjuvant group (N=43)

**Pathological feature**	**45M1**	**CLH2**	**EC**	**EC-45M1 CS**
Pathological differentiation, G1-2 vs. G3	0.8699	0.9096	0.6690	0.4631
Peripancreatic extension	0.3616	0.1590	0.8970	0.7297
Treatment effect, OR vs. NR	0.1842	0.1129	0.6558	0.5419
Lymphovascular invasion	0.6087	0.7198	0.9769	0.8538
Perineural invasion	**0.0419**	*0.0662*	0.5529	0.1203
Margins	*0.0707*	0.1182	0.1855	0.2252
R0 vs R1-2	**0.0300**	*0.0811*	*0.0710*	*0.0658*
Tumor size (≤ 2 cms vs. > 2cm)	0.2123	0.1614	0.4538	0.1706
N0 vs N1-N1	0.7511	0.6899	0.4089	0.4653
Premalignant, yes vs. no	0.7749	0.9116	**0.0136**	0.2844
Neoadjuvant CRT	0.9160	0.9457	0.6558	0.7519
Site of recurrence (none vs. local vs. distant)	0.3041	0.3106	0.7705	0.5442

G-grade, OR-objective response, NR- no response, UpS – upfront surgery, EC-extracellular, CS – composite score, CRT – chemoradiation

Supplementary Table S4: Multivariate analysis of clinicopathological features for progression-free survival in the FOLFIRINOX neoadjuvant group (N=36)

**Parameter**		**Pr > ChiSq**	**Hazard Ratio**	**95% Wald Confidence Limits**
**45M1 - Hscore**		**0.0145**	**0.944**	**0.901-0.989**
CLH2- H-score		***0.0597***	***1.042***	***0.998 – 1.088***
**EC-detection**	**Negative**	**0.0282**	**0.210**	**0.05 – 0.847**
Pathological differentiation, G1-2 vs G3	G1-2	0.1853	0.450	
Peripancreatic extension	NO	0.4372	1.749	
Treatment effect OR vs. NR	OR	0.2947	0.426	
**Lymphovascular invasion**	**NO**	**0.0022**	**0.080**	**0.016 – 0.404**
Perineural invasion	NO	0.4661	0.523	
**Margins**	**Negative**	**<.0001**	**0.035**	**0.007 – 0.185**
R0 vs R1-R2	R0	0.1080	0.188	
Tumor size (≤ 2 cms vs. > 2cm)	≤ 2 cms	0.2618	0.282	
N0 vs N1-N1	N0	0.1856	3.647	
**Premalignant**	**None**	**0.0151**	**0.107**	**0.018- 0.649**
NAT CRT	Had NAT CRT	0.9238	0.937	
**Site of recurrence**	**Distant metastasis[1]**	**0.0049**	**18.767**	**2.44 – 144.9**
**Site of recurrence**	**Local recurrence[1]**	**0.0066**	**18.860**	**2.265 – 157.067**

1- vs. no recurrence, G-grade, OR-objective response, NR- no response, EC-extracellular, CS – composite score, CRT – chemoradiation, NAT- neoadjuvant therapy

Supplementary Table S5: Multivariate analysis of clinicopathological features for overall survival in the FOLFIRINOX neoadjuvant group (N=36)

**Parameter**		**Pr > ChiSq**	**Hazard Ratio**	**95% Wald Confidence Limits**
**45M1 – H score**		**0.0005**	**0.867**	**0.801-0.939**

**CLH2- H score**		**0.0014**	**1.127**	**1.047 – 1.212**
EC-detection	Negative	0.4486	0.493	
**Pathological differentiation, G1-2 vs G3**	**G1-2**	**0.0020**	**0.067**	**0.012- 0.372**
**Peripancreatic extension**	**NO**	**0.0192**	**5.526**	**1.321- 23.114**
Treatment effect OR vs. NR	OR[1]	0.8928	1.144	
**Lymphovascular invasion**	**NO**	**0.0020**	**0.075**	**0.015 – 0.388**
Perineural invasion	NO	0.3999	0.455	
**Margins**	**Negative**	**<.0001**	**0.003**	**0.000-0.038**
R0 vs. R1-R2	R0	0.2216	0.259	
**Tumor size (≤ 2 cms vs. > 2cm)**	**≤ 2 cms**	**0.0191**	**0.027**	**0.001-0.553**
N0 vs. N1-N1	N0	0.2112	3.600	
**Premalignant**	**None**	**0.0030**	**0.050**	**0.007-0.362**
NAT CRT	**Had NAT CRT**	*0.0769*	*3.255*	*0.880-12.034*
**Site of recurrence**	**Distant metastasis[1]**	**0.0155**	**11.751**	**1.59- 86.353**
Site of recurrence	Local recurrence[1]	0.2640	3.405	

1-vs. No response; 2- vs. no recurrence, G-grade, OR-objective response, NR- no response, UpS – upfront surgery, EC-extracellular, CS – composite score, CRT – chemoradiation, NAT – neoadjuvant therapy

Supplementary Table S6: Multivariate analysis of clinicopathological features for progression-free survival in the gemcitabine-based adjuvant therapy in the upfront surgery group (N=57)

**Parameter**		**Pr > ChiSq**	**Hazard Ratio**	**95% Wald Confidence Limits**
**45M1 – H score**		**0.0301**	**1.030**	**1.003 – 1.058**
CLH2- H score		*0.0735*	*0.977*	*0.953-1.002*
EC-detection	Negative	0.3697	1.766	
Pathological differentiation, G1-2 vs G3	G1-2	0.7515	0.862	

Peripancreatic extension	NO	0.5512	1.312	
**Lymphovascular invasion**	**NO**	**0.0376**	**0.210**	**0.048-0.914**
**Perineural invasion**	**NO**	**0.0431**	**0.152**	0.025-0.944
Margins	Negative	0.4263	0.471	
R0 vs R1-R2	R0	0.7279	1.444	
Tumor size (≤ 2 cms vs. > 2cm)	≤ 2 cms	0.2921	0.522	
N0 vs N1-N1	N0	0.1275	0.283	
Premalignant	None	0.6062	0.772	
**Site of recurrence**	**Distant metastasis**[1]	**0.0102**	**10.379**	**1.74 – 61.884**
**Site of recurrence**	**Local recurrence**[1]	**0.0001**	**60.912**	**7.348 – 504.95**

1-vs. No response; 2- vs. no recurrence, G-grade, OR-objective response, NR- no response, UpS – upfront surgery, EC-extracellular, CS – composite score, CRT – chemoradiation

Supplementary Table S7: Multivariate and univariate analysis of clinicopathological features for progression-free survival and overall survival in various groups

Pathological feature	Significant impact (p<0.05)	
	PFS	OS
Gem-based group - MVA	Lymphovascular invasion (LVI)<br>Perineural invasion (PNI)<br>Site of recurrence- local and distant	Site of recurrence – local and distant
Gem-based group - UVA	PPEperipancreatic extension (PPE)<br>LVI<br>Margins<br>Residual disease (Rd, Ro vs. R1-R2)<br>Lymph node-positive (LN+, N0 vs. N1-N1)<br>Site of recurrence distant and local	PPE<br>LVI<br>Margins<br>Rd<br>LN+<br>Site of recurrence distant and local
Gem-only group - MVA	PPE<br>LVI<br>Site of recurrence local	PPE<br>LVI<br>Site of recurrence distant and local

Gem-only group- UVA	LVI<br>PNI<br>LN+<br>Site of recurrence distant and local	LVI<br>PNI<br>Margins<br>Rd<br>LN+<br>Site of recurrence distant and local
All patients - MVA	Treatment response<br>LVI<br>Site of recurrence<br>Margins<br>NAT-CRT	Grade<br>PPE<br>TR<br>Site of rec
All patients - UVA	PPE<br>TR<br>LVI<br>PNI<br>Margins<br>R0-R1-2<br>NAT-CRT<br>NAT<br>Site of recurrence distant and local	Grade<br>PPE<br>TR<br>LVI<br>PNI<br>Margins<br>R0-R1-2<br>NAT-CRT<br>NAT<br>Site of recurrence distant and local
Upfront surgery group -MVA	Site of recurrence distant local<br>Premalignant	Distant mets<br>Premalignant<br>Site of recurrence distant and local
Upfront surgery group -UVA	PPE<br>LVI<br>Margins<br>Rd<br>LN+<br>Site of recurrence distant and local	PPE<br>LVI<br>Margins<br>Rd<br>LN+<br>Site of recurrence distant and local

MVA – multivariate analysis, UVA – univariate analysis, Gem- gemcitabine based including combinations of capecitabine, nab-paclitaxel, and Gem-alone

Supplementary Table S8: Site of metastasis and corresponding biopsy sites tissue provided in the study.

Distant metastases (N)	Biopsy site provided for MUC5AC testing.
Liver (3)	Liver
Liver (1)	Pancreas
Lung (1)	Lung
Liver, peritoneum, and ovary (1)	Ovary
Peritoneum and small bowel (2)	Peritoneal lesion -1<br>Small bowel -1
Peritoneum (1)	Peritoneal lesion
Lung and adrenal gland with PDA invading the bladder (1)	Bladder

Supplementary Table S9: Comparing MUC5AC expression between metastatic and primary tumors.

	45M1	CLH2	EC-45M1
	Metastatic sites (N=9) vs. resected primary sites (N=100) (p-value)		
H-score	*199 vs. 149 (0.05)*	**196 vs. 145 (0.04)**	**199 vs. 132 (0.02)***
% distribution	**68 vs. 50 (0.03)**	**67 vs. 50 (0.03)**	NA
Detection rate	100 vs. 96#	100 vs. 96#	*100 vs. 72 (0.06)*

NA- not applicable

Supplementary Table S10: Comparing MUC5AC distribution in primary and metastatic tissues

	Treated (n=8) vs. UpS (N=57)	Treated (N=8) vs. NAT-group (N=43)	Mets= treated vs. not treated
45M1	*225 vs. 164 (0.05)*	**225 vs. 128 (0.006)**	**225 vs. 115 (0.04)**
CLH2	**221 vs. 163 (0.043)**	**221 vs. 121 (0.005)**	**221 vs. 115 (0.04)**
EC-45M1	**225 vs. 150 (0.03)**	**225 vs. 108 (0.03)**	**225 vs. 115 (0.04)**
EC-detection %	100 vs. 82 (0.1)	**100 vs. 58 (0.02)**	

Ups – upfront surgery, NAT – neoadjuvant therapy, EC- extracellular

Supplementary Table S11: **Breakdown of neoadjuvant therapy group (N=43) based on treatment**

	**Objective response vs. no response group[P]<br>H-score mean**		
	45M1	EC-45M1*	CLH2
FOLFIRINOX (36)<br>FOLFOX (2)#<br>Gem-NP (5)	110 vs.153<br>240<br>140 vs. 80	54 vs. 58<br>100<br>67 vs. 50	105 vs. 149<br>240<br>97 vs. 80
5FU-based<br>Gem-based	110 vs. 165<br>140 vs. 80	54 vs. 64<br>67 vs. 50	105 vs. 165<br>97 vs. 80

P = p value of all the comparisons were > 0.1. *Detection rates, # two patients had NR, Gem-gemcitabine, 5FU-5flurouracil, NP- nab-paclitaxel, Ups – upfront surgery

Supplementary Figure S1: Breakdown of the samples used in the project.

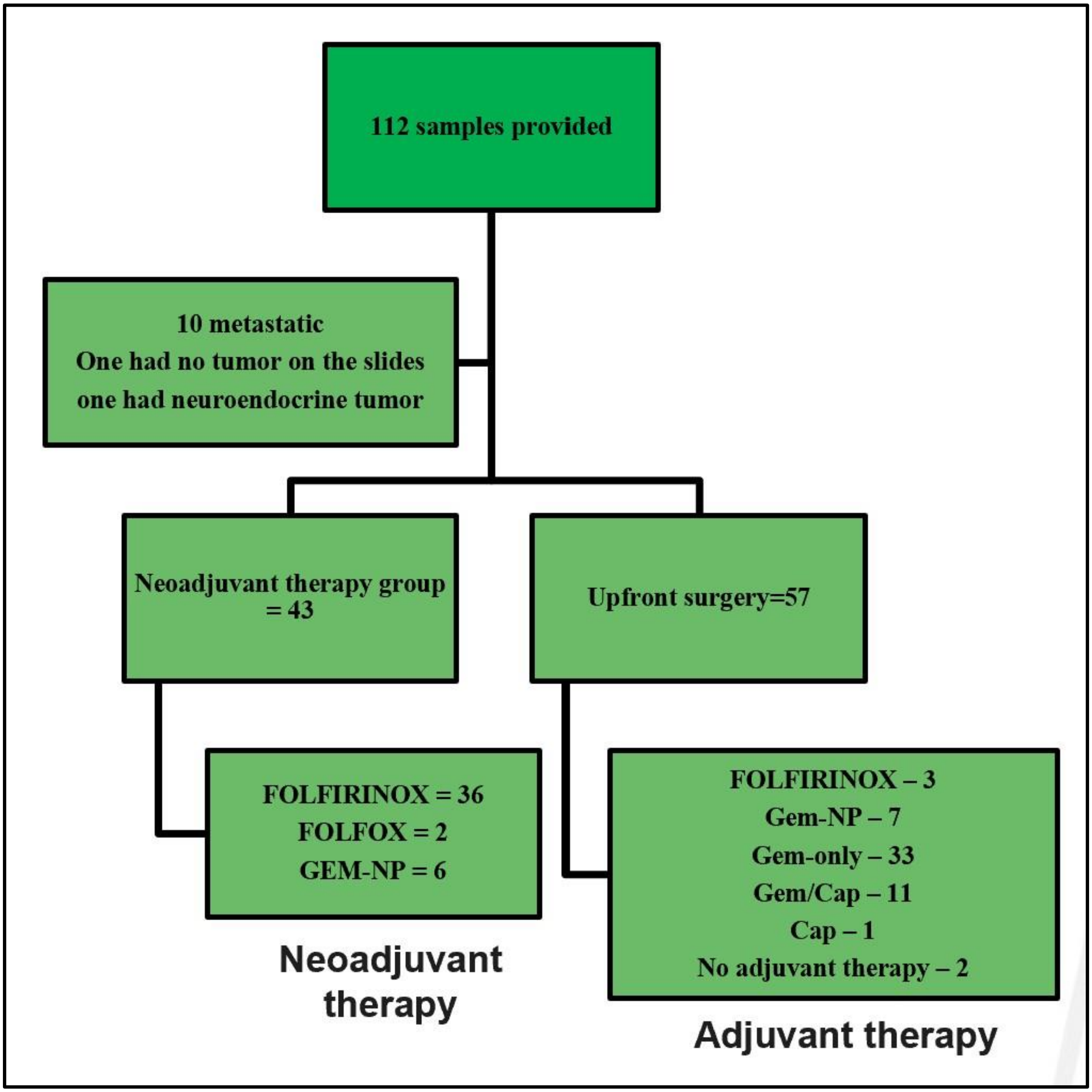

Gem-gemcitabine, NP – nab/paclitaxel

Supplementary Figure S2: MUC5AC expression in pancreatic ductal adenocarcinoma

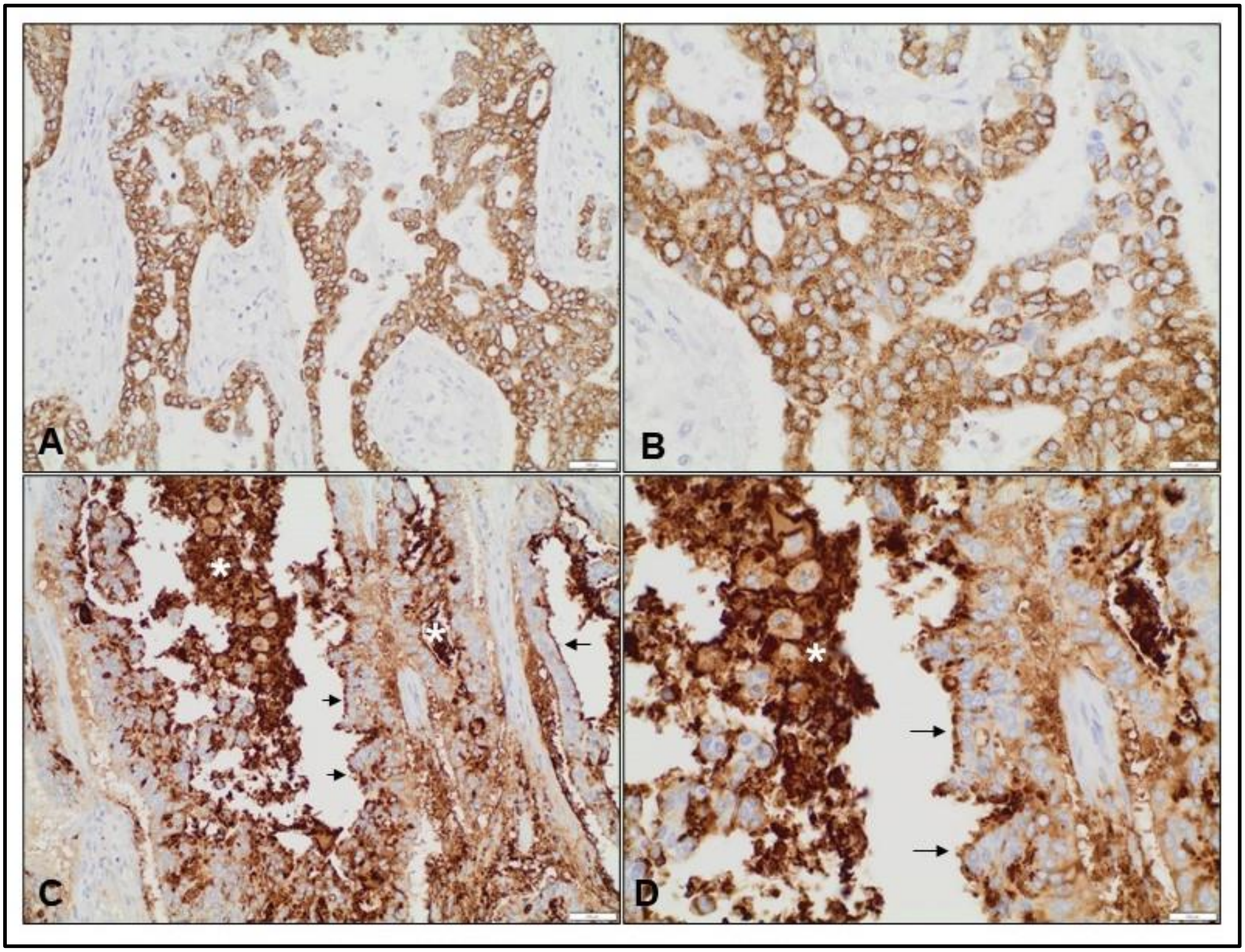

Pancreatic ductal adenocarcinoma with strong cytoplasmic expression for CLH2 without extracellular expression (A-200X, and B-400X). Same tumor showing strong apical expression (black arrow) and extracellular staining (*) for 45M1 (C-200X and D-400X).

Supplementary Figure S3: Impact of neoadjuvant therapy on median survival in the study population.

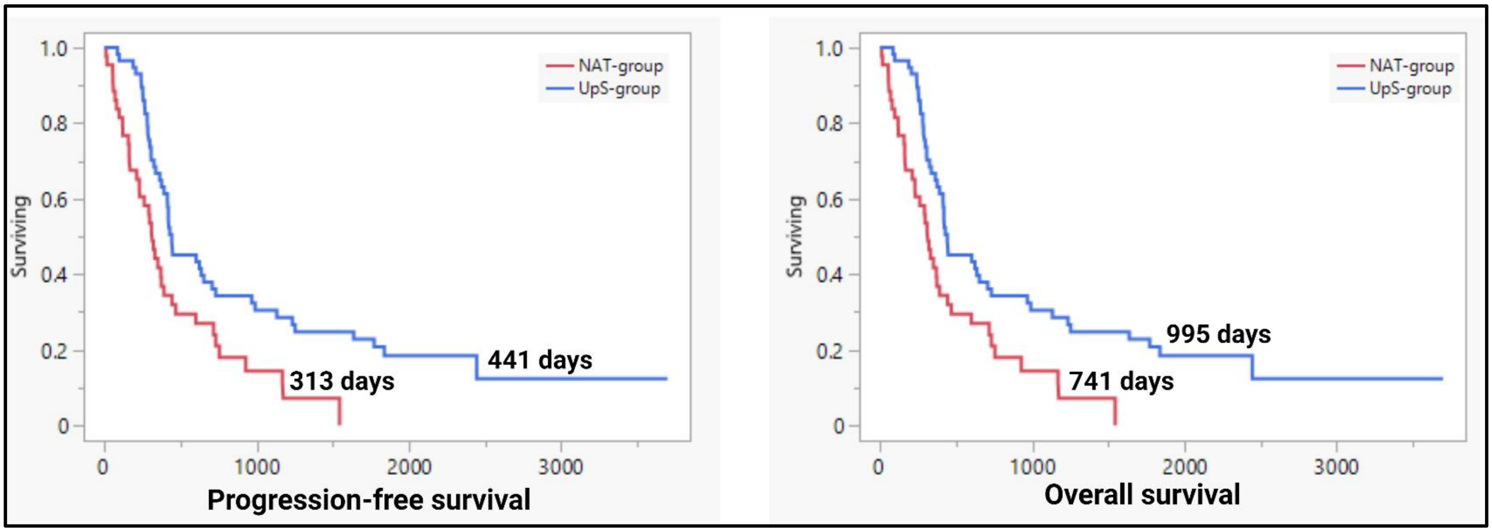

NAT-neoadjuvant therapy; UpS- upfront surgery.
